# Expression of neuropeptide Y is increased in an activated human HSC cell line

**DOI:** 10.1038/s41598-019-45932-3

**Published:** 2019-07-01

**Authors:** Wufei Dai, Yang Liu, Yali Zhang, Yufeng Sun, Changjiang Sun, Yu Zhang, Xiufang Lv

**Affiliations:** 1 Basic Medical Research Centre, Medical College of Nantong University, Nantong, China; 2 Department of Biochemistry, Medical College of Nantong University, Nantong, China; 3 Department of Clinical Laboratory, Affiliated Hospital of Nantong University, Nantong, China; 4 Department of hepatobiliary Surgery, Affiliated Hospital of Nantong University, Nantong, China

**Keywords:** Predictive markers, Predictive markers, Hepatology, Hepatology

## Abstract

Neuropeptide Y (NPY) is an abundant neuropeptide in the mammalian central and peripheral nervous systems. Transgenic mice overexpressing NPY in noradrenergic neurons have increased level of hepatic triglycerides, fatty acids and cholesterol, which contributed to the development of hepatosteatosis. However, the roles of NPY in the activation of hepatic stellate cells (HSCs) and the underlying mechanisms remain unclear. This study aimed to investigate the expression and secretion of NPY in human immortalized HSC LX-2 cells and the regulatory function of NPY on the fibrogenic response in LX-2 cells, to explore the potential association between NPY and LX-2 activation. The results showed an increase in the expression and secretion of NPY(1–36) in activated LX-2 cells. Both endogenous and exogenous NPY(1–36) induced the phosphorylation of mTOR, p70S6K, and 4EBP1 and promoted the fibrogenic response via NPY Y1 receptor subtype (NPY1R), as these responses were blocked by either an NPY1R antagonist (BIBP3226) or NPY1R knockdown. Moreover, NPY(1–36) serum levels were increased in patients with liver cirrhosis (LC) and hepatocellular carcinoma (HCC) and presented a positive relationship with MELD scores in LC patients. These findings suggest that immortalized HSCs LX-2 have the potential to produce NPY(1–36). High serum levels of NPY(1–36) is correlated with hepatic dysfunction in cirrhotic patients.

## Introduction

Neuropeptide Y (NPY) is the most abundant neuropeptide in the mammalian central nervous system and plays important roles in feeding behaviours, depression, stress, and hypothalamic hormone release^1^. In the peripheral nervous system, NPY is co-exists with norepinephrine in nerve endings in the sympathetic nerve system. It is a regulator of the cardiovascular system and a growth factor in endothelial cells and vascular smooth muscle cells (VSMCs)^2^. NPY has also been implicated as a promotor of multiple types of malignancies, including breast and prostate cancer^3,4^. Moreover, several studies have shown that transgenic mice overexpressing NPY in noradrenergic neurons show increased hepatic level of triglycerides, fatty acids and cholesterol, which contributed to the development of hepatosteatosis^5^. Whether NPY is involved in HSCs activation, fibroblastic changes and the underlying mechanisms are unclear.

Hepatic fibrosis and cirrhosis are the result of multiple types of liver injury caused by various factors, including viruses, metabolic diseases and cholestatic, autoimmune and drug induction^6^. Many studies both in animal models and in humans has shown that fibrogenesis is a dynamic and reversible process^7,8^. Underlying this process is a variety of nonparenchymal liver cells that transdifferentiate into activated cells characterized by increased cell proliferation and extracellular matrix secretion. Cirrhosis represents an advanced stage of fibrosis, which is present in 80–90% of patients with hepatocellular carcinoma (HCC)^9,10^. Thus, the elucidation of the mechanisms underlying these processes is fundamentally important for developing effective antifibrotic therapies and decreasing the risk of HCC among patients.

Liver myofibroblasts arising from different cell populations, such as resident mesenchymal cells, portal fibroblasts, and hepatic stellate cells (HSCs), undergo a common process of transdifferentiation^6,11^. Among these cell populations, HSCs are the main source of myofibroblasts (>80%)^12,13^. In liver tissue, HSCs are located in the space between hepatocytes and liver sinusoidal endothelial cells and represent 5–8% of the total number of hepatic cells. HSCs are in contact with a large number of hepatocytes, adjacent sinusoidal endothelial cells, stellate cells, and nerve endings. They are important autocrine, paracrine, and chemoattractant factors that are used to maintain microenvironment homeostasis in the hepatic sinusoid^14^. After acute or chronic liver injury, HSCs transdifferentiate from retinoid storing cells to activated myofibroblast-like cells characterized by the expression of α-smooth muscle actin (α-SMA) and the loss of retinoid. The process of activation includes myofibroblast-like cell phenotypic changes, including increased migration capability and proliferation rate increasing, increased production of cytokines, chemokines and extracellular matrix proteins, and enhanced contractility, which may lead to fibrosis and cirrhosis^15^.

We previously demonstrated that NPY inhibits the proliferation of human immortalized hepatocyte cells and HCC cells^16^. On one hand, NPY-positive nerve fibres are localized in Disse spaces in very close proximity to HSCs and adjacent hepatocytes^17^. On the other hand, HSCs contact a large number of nerve endings through multiple cytoplasmic processes. Nevertheless, limited information exists regarding the relationship between NPY and HSCs activation, the fibrogeneic response, and even hepatic fibrosis. Thus, the aims of this study were to assess the expression and function of NPY in immortalized LX-2 HSCs, to explore the signalling pathways involved and to obtain *in vitro* experimental evidence of the fibrogenic response to NPY in the human HSC cell line. Moreover, we sought to investigate human circulating NPY levels and their relationship with the severity of liver cirrhosis.

## Results

### Expression of NPY and NPY1R in human immortalized LX-2 HSCs and liver tissue

**Figure 1:**
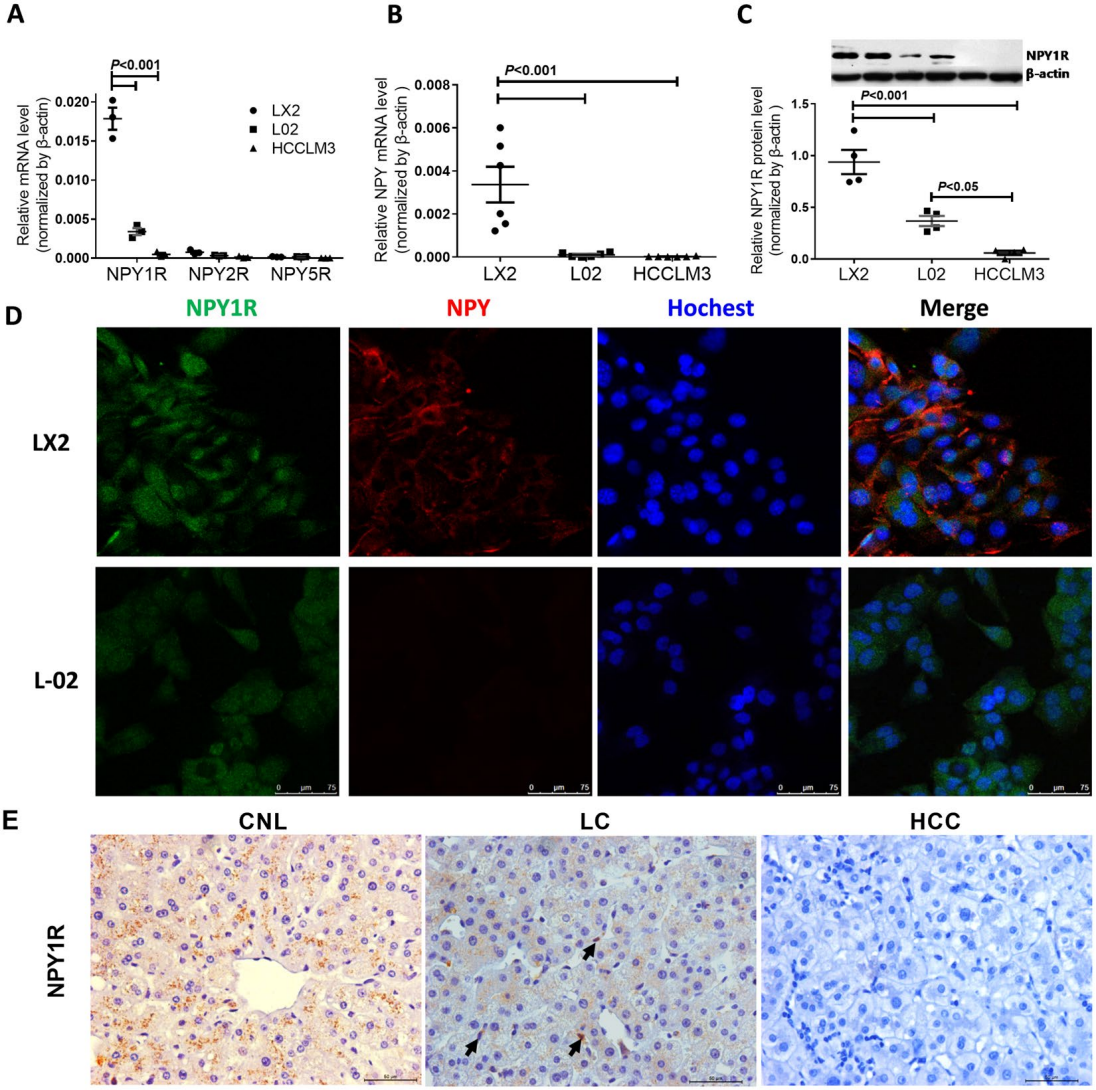
Expression of NPY and its receptors in cultured cells and liver tissues. (**A**) mRNA expression of NPY1R, NPY2R and NPY5R in LX-2, L-02 and HCCLM3 cells. (**B**) NPY mRNA expression in LX-2, L-02 and HCCLM3 cells. (**C**) Protein levels of NPY1R in LX-2, L-02 and HCCLM3 cells. The lanes represent two duplicate samples of each cell type. (**D**) Representative images showing the distribution of NPY (red) and NPY1R (green) in LX-2 and L-02 cells. The nuclei are stained with Hoechst (blue). Scale bars, 75 µm. (**E**) Representative images of NPY1R immunostaining in liver tissues. The black arrows indicate HSCs. CNL, control normal liver; LC, liver cirrhosis; HCC, hepatocellular carcinoma. Scale bars, 50 µm.

Our previous study demonstrated that the levels of NPY1R mRNA and protein in human immortalized hepatocyte L-02 cells were significantly higher than those in HCC cells. Until now, there have been limited data demonstrating the potential for HSCs to express NPY receptors and to synthesize NPY. Here, we demonstrated that mRNA encoding NPY1R, but not NPY2R or NPY5R, was expressed more strongly in LX-2 cells than in L-02 cells and HCCLM3 cells (Fig. 1A). The level of NPY mRNA in LX-2 cells was significantly higher than that in L-02 cells and HCCLM3 cells. Importantly, almost no NPY mRNA was detected in L-02 cells or HCCLM3 cells (Fig. 1B). The NPY1R protein level, as determined by western blotting, was the highest in LX-2 cells among the three cell lines (Fig. 1C). We further demonstrated via fluorescence microscopy that both LX-2 cells and L-02 cells expressed the NPY1R protein and that it was diffusely spread throughout the cell cytoplasm and nucleus (green). LX-2 cells, but not L-02 cells, abundantly expressed the NPY protein only in the cytoplasm (red) (Fig. 1D). Neither NPY nor the NPY1R protein was detected in HCCLM3 cells (data not shown). These results suggest that NPY and NPY1R are expressed in immortalized LX-2 HSCs.

Based on the above, we wanted to know which cells express NPY and its receptors in human liver tissue. We analysed the expression of NPY1R, NPY2R, NPY5R and their common agonist NPY in clinical samples by immunohistochemical (IHC) staining. The results showed that NPY1R was mainly located in the cytoplasm and there was low expression on the membranes of hepatocytes in control normal livers (CNLs), weak staining in fibrotic livers (LCs) and no staining in hepatocellular carcinoma (HCC) tissues. What excited us was that the NPY1R staining was stronger in HSCs than in the hepatocytes in LC tissues (Fig. 1E). NPY2R and NPY5R were not detected in any tissues (data not shown). We failed to detect the NPY protein in HSCs and hepatocytes. The pattern of staining was faint cytoplasmic staining in some stromal cells near the parenchymal-stromal inter-face in CNL tissues (Figure S1). These results suggest that NPY1R, but not NPY2R and NPY5R, are expressed in the human liver. Our data indicate that NPY1R is the prominent NPY receptor subtype expressed in LX-2 cells and human liver tissues. Therefore, we focused only on the NPY(1–36), the ligand with the highest affinity among the various isoforms, in subsequent studies.

### Human immortalized HSCs LX-2 synthesize and secrete NPY(1–36)

**Figure 2:**
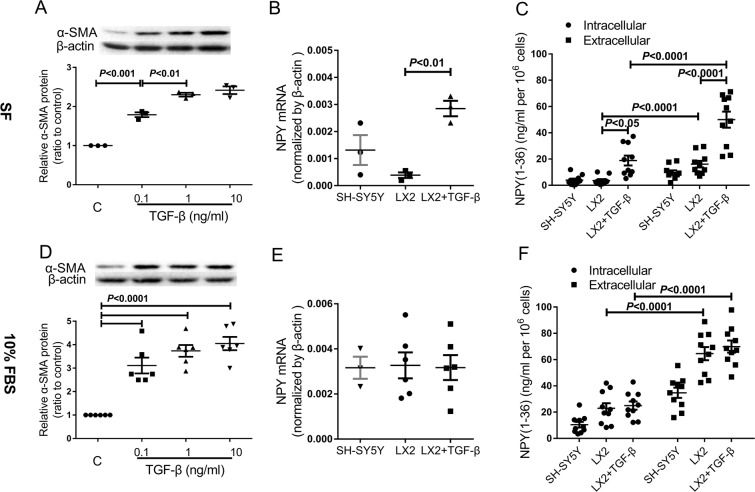
Expression and secretion of NPY(1–36) in activated LX-2 cells. (**A**) α-SMA protein levels in TGF-β1-treated LX-2 cells cultured in SF medium. (**B**) NPY(1–36) mRNA expression in SH-SY5Y cells and LX-2 cells with or without TGF-β1 (1 ng/ml) treatment cultured in SF medium. (**C**) NPY(1–36) protein concentration inside cells (intracellular) and released into the culture medium (extracellular) when cells were cultured in SF medium. (**D**) α-SMA protein levels in TGF-β1-treated LX-2 cells cultured in medium containing 10% serum. (**E**) NPY(1-36) mRNA expression in SH-SY5Y cells and LX-2 cells with or without TGF-β1 (1 ng/ml) treatment cultured in 10% FBS medium. (**F**) Intracellular and extracellular NPY(1–36) protein concentration of cells cultured in 10% FBS medium.

LX-2 cells exhibit typical features of hepatic stellate cells and express α-SMA under culture conditions, even when grown in low serum mediums (1% FBS). Therefore, the cells are considered activated, or at least partially activated. NPY(1–36) mRNA and protein were present in LX-2 cells cultured in 10% FBS, and the response of the LX-2 cells to TGF-β1 involved accelerating HSC activation. We wanted to determine whether the NPY(1–36) protein can be synthesized in LX-2 cells and secreted by the cells to play important regulatory functions. To assess NPY(1–36) synthesis and secretion in activated HSCs, LX-2 cells were cultured in serum-free medium with or without TGF-β1 treatment for 24 h. Human SH-SY5Y neuroblastoma cells, which have been reported to express and secrete NPY(1–36), were used as a positive control^18^. First, we examined the protein expression of the activated HSC marker α-SMA in the TGF-β1-treated cells. Quantitative analysis showed that the α-SMA protein was significantly upregulated in treated cells cultured in serum-free (SF) medium by three doses (0.1, 1 and 10 ng/mL) of TGF-β1 (Fig. 2A). We chose a dose of 1 ng/mL for subsequent TGF-β1 treatment studies. To investigate NPY(1–36) synthesis and secretion in activated LX-2 cells, we examined NPY(1–36) mRNA and protein expression in TGF-β1-treated LX-2 cells. As shown in Fig. 2B,C, TGF-β1 significantly enhanced the NPY(1–36) mRNA level in LX-2 cells cultured in serum-free medium. NPY(1–36) protein concentrations in both LX-2 cell lysates (intracellular) and SF medium (extracellular) were significantly enhanced by TGF-β1 (Fig. 2C). These results suggest that TGF-β1-induced LX-2 activation leads to the synthesis and secretion of the NPY(1–36) protein by LX-2 cells.

A previous study showed that 10% FBS induced HSCs proliferation. There are reasons to believe that LX-2 cells undergo progressive activation with serum growth factor support^19^. Therefore, it was of interest to investigate the effects of 10% FBS medium on TGF-β1-induced LX-2 cell activation and the pattern of NPY(1–36) expression and secretion. We cultured LX-2 cells for 24 h in 10% FBS medium with or without TGF-β1 treatment. Western blot analysis demonstrated that TGF-β1 markedly induced α-SMA protein expression at three doses, and there was no difference among the three doses (Fig. 2D). TGF-β1 had no effect on NPY(1–36) mRNA expression (Fig. 2E) or changes in protein concentration in LX-2 cell lysates (intracellular) or in culture medium (extracellular) (Fig. 2F). Together, these results indicate that TGF-β1 promoted further activation of LX-2 cells to produce α-SMA protein but had no effect on the synthesis and secretion of NPY(1–36) protein in already activated LX-2 cells.

### NPY(1–36) promotes LX-2 cell proliferation and migration via NPY1R

**Figure 3:**
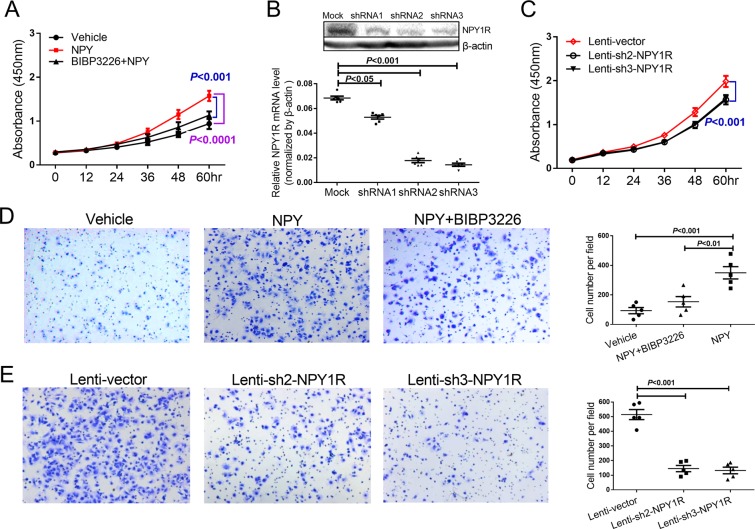
Effect of NPY(1–36) on LX-2 cell proliferation and migration via NPY1R. (**A**) Cell proliferation was analysed by treatment with NPY(1–36) (10^−8^ M) or BIBP3226 (10^−7^ M) pretreatment. (**B**) NPY1R was knockdown by shRNA in stable Lenti-sh2-NPY1R and Lenti-sh3-NPY1R transfectants. (**C**) Cell proliferation was analysed by NPY1R knockdown. (**D**) Representative cell migration images treated with vehicle, NPY(1–36),or NPY(1–36) with BIBP3226 pretreatment. The histogram is the statistical analysis of cell counts. (**E**) Representative cell migration images of NPY1R knockdown. The histogram is the statistical analysis of cell counts.

The effects of HSC activation, including cell proliferation and migration, can be considered a fibrogenic response to stimuli present in the extracellular environment. As LX-2 cells synthesize and secrete NPY(1–36) and obviously express one of its receptors, namely, NPY1R, we wanted to determine whether the cells respond functionally to NPY(1–36) via NPY1R. We treated LX-2 cells with human recombinant NPY(1–36) in serum-free culture medium with or without pretreatment with the selective NPY1R antagonist BIBP3226. Our data showed that NPY(1–36) significantly promoted the fibrogenic response, such as cell proliferation and migration in LX-2 cells. Pre-treatment with BIBP3226 blocked the promotion effect of NPY(1–36) (Fig. 3A,D). There is limited knowledge about the function of endogenous NPY(1–36) in HSCs. To explore the biological function of endogenous NPY(1–36) and NPY1R in HSCs, we established stable cell lines transduced with a lentivirus carrying short hairpin RNA (shRNA) targeting NPY1R, as described previously^16^. NPY1R was successfully reduced in LX-2 cells stablely transfected with either Lenti-sh2-NPY1R or Lenti-sh3-NPY1R (Fig. 3B). Then, we cultured these stably transfected cells in medium containing 10% FBS and assessed cell proliferation and migration. Our data showed that NPY1R was a key factor involved in proliferation and migration and that the knockdown of NPY1R significantly inhibited the basal proliferation and migration of the LX-2-sh-NPY1R-transfectanted cells (Fig. 3C,E). These results suggest that NPY(1–36) functions as an activator to promote fibrogenic response on LX-2 cells via NPY1R.

### NPY(1–36) induces LX-2 cell activation through the mTOR/p70S6K/4EBP1 signalling pathway

**Figure 4:**
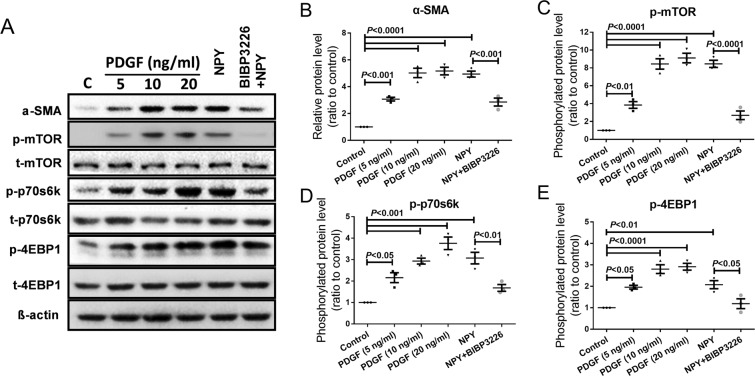
Phosphorylation of mTOR, p70S6K and 4EBP1 in LX-2 cell. (**A**) Representative images of phosphorylated mTOR, p70S6K and 4EBP1 were determined in cell lysates by western blotting. Cells treated with various dosages of PDGF as a positive control. Quantification of α-SMA protein (**B**), phosphorylation of mTOR (**C**), p70S6K (**D**) and 4EBP1(**E**) protein in NPY-treated cells, with or without pretreatment of BIBP3226.

The mammalian target of rapamycin (mTOR) pathway plays key roles in numerous cellular functions, including the transdifferentiation of HSCs in liver fibrosis^20,21^. Both the 70-kDa ribosomal S6 kinase (p70S6K) and eukaryotic translation initiation factor 4E binding protein 1 (4EBP1) are downstream molecules of mTOR. To evaluate whether the mTOR signalling pathway is involved in NPY-induced LX-2 cell activation and if this activation occurs via NPY1R, LX-2 cells were treated with NPY(1–36) for 24 h with or without BIBP3226 following serum starvation for 48 h. PDGF-treated LX-2 cells were used as a positive control^22^. To examine the effect of NPY(1–36) on signal transduction in LX-2 cells, we performed western blot analysis with specific phosphoantibodies on cell lysates from activated LX-2 cells. The results showed that PDGF induced α-SMA expression and the phosphorylation of mTOR, p70S6K and 4EBP1, as expected. α-SMA was significantly induced by NPY(1–36), and this induction was abolished by BIBP3226 pretreatment (Fig. 4A,B). The phosphorylation of mTOR, p70S6K and 4EBP1 was significantly increased by NPY(1–36) treatment (Fig. 4A,C–E). This effect shows specificity via NPY1R because the activation of mTOR, p70S6K and 4EBP1 was significantly decreased by pretreatment with BIBP3226 (Fig. 4A,C–E). These data collectively indicate that the activation of the mTOR/p70S6K/4EBP1 pathway is necessary for the NPY-stimulated fibrogenic response in LX-2 cells via NPY1R.

### Serum levels of NPY(1–36) correlate with liver dysfunction in human hepatic cirrhosis

**Figure 5:**
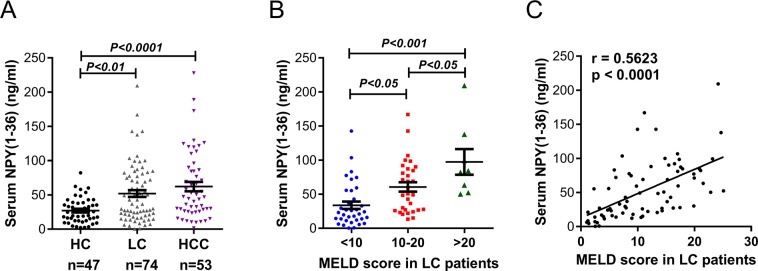
NPY(1–36) serum levels in patients with liver cirrhosis and HCC. (**A**) NPY(1–36) serum levels in HC and patients with LC and HCC. (**B**) NPY(1–36) serum levels in LC patients with different MELD scores. (**C**) In LC patients, correlation analysis between changes in the NPY(1–36) serum concentration and MELD scores (r = 0.5725, *P* < 0.0001). HC, healthy control; LC, liver cirrhosis; HCC, hepatocellular carcinoma.

Because our results suggest the importance of NPY(1–36) expression, secretion and function in LX-2 cell activation, we next attempted to determine whether there are changes in the circulating NPY(1–36) concentration in patients with different liver diseases. Therefore, we determined the NPY(1–36) concentration in serum samples from LC and HCC patients by ELISA and compared them to NPY(1–36) levels in healthy control (HC) individuals. As shown in Fig. 5A, the serum level of NPY(1–36) was significantly increased in both patients with LC and HCC when compared to healthy individuals, but no differences were observed between patients with LC and HCC. To further confirm these changes, we carried out Pearson’s chi-square and Fisher’s exact test to analyse the correlation between NPY(1–36) concentrations and clinicopathological characteristics in patients. The same result as that observed above was revealed (Table S5).

In order to identify the NPY(1–36) serum level is associated with the severity of liver cirrhosis, we adopted a scoring system named the Model for End-Stage Liver Disease (MELD) to assess the severity of chronic liver disease and to evaluate hepatic function in cirrhotic patients^23,24^. Patients were divided into three groups in terms of MELD scores: < 10, 10-20, and > 20. A stepwise increasing trend in NPY(1–36) serum concentrations was found in LC patients as MELD scores increased (Fig. 5B). We further analysed the correlation between circulating NPY(1–36) levels and MELD scores. As shown in Fig. 5C, Pearson correlation analysis revealed a remarkable positive correlation between NPY(1–36) serum concentration and MELD score (R^2^ = 0.3162, p < 0.0001, r = 0.5623). These findings underscore the relevance of circulating NPY(1–36) levels to the developing of liver cirrhosis in HCC.

## Discussion

HSCs are the main source of liver myofibroblasts, and recent research has suggested that activated HSCs should be regarded as the primary target for developing new antifibrotic therapies^25^. LX-2 is an immortalized human hepatic stellate cell line that is usually used as a cell model to study the mechanism of liver fibrogenesis and to test the antifibrotic effect of new compounds^26,27^. In our present study, we found for the first time that activated human immortalized hepatic LX-2 cells mainly expressed NPY1R, but not NPY2R and NPY5R, and secreted NPY(1–36). After activated LX-2 cells were quiesced by serum starvation, TGF-β1 and medium containing 10% FBS activated the quiescent HSCs and induced the expression and secretion of NPY(1–36). Nevertheless, TGF-β1 had no effect on NPY(1–36) expression and secretion in activated LX-2 cells. The findings that NPY1R is expressed and NPY2R and NPY5R are absence in LX-2 cells are similar to Sigala’s findings that primary human HSCs abundantly express the NPY Y1 receptor subtype but exhibit little or no expression of the Y2 and Y5 subtypes^28^.

Our previous study found that NPY(1–36) inhibits human HCC cell proliferation *in vitro*, while other findings have shown that NPY increases the proliferation of cultured primary human HSCs^28,29^. Similar to other reports, the present study found that human immortalized HSCs LX-2 synthesize and secrete NPY(1–36). This endogenous NPY(1–36) promoted a fibrogenic response to induce the proliferation and migration of LX-2 cells, as did exogenous recombinant human NPY(1–36). We also found that NPY-induced proliferation and migration occurred through NPY1R because these responses were blocked either by BIBP3226 pretreatment or by NPY1R knockdown. However, little is known about the possible intracellular pathway-mediated effects of NPY(1–36). Some findings have shown that the phosphorylation of mTOR and p70S6K is induced in activated HSCs and regulates HSCs proliferation and migration^30,31^. Moreover, a number of mTOR inhibitors have been reported to inhibit HSC proliferation and attenuate hepatic fibrosis *in vivo* and *in vitro*. Similar to previous studies, our data showed that NPY(1-36) induced the phosphorylation of mTOR and p70S6K, as well as that of their common substrate 4EBP1, which functions as a translation repressor and is involved in the regulation of LX-2 cell activation^32^. In the hypophosphorylated form, 4EBP1 binds to eIF4E to prevent cap-dependent translation^33,34^. In the present study, upon stimulation by NPY(1–36), phosphorylated mTOR/p70S6K induced the phosphorylation of 4EBP1, leading to translation initiation and the subsequent proliferation and migration of LX-2 cells.

Transgenic mice overexpressing NPY exhibit increased levels of hepatic triglycerides, fatty acids and cholesterol, which contribute to the development of hepatosteatosis^5^. Another study showed that stress-induced increases in serum levels of NPY combined with a high-fat and high-sugar (HFS) diet leads to liver steatosis by promoting fat growth^35^. These data suggest that NPY may be involved in fibroblastic changes in liver fibrosis and cirrhosis, particularly because hepatosteatosis is the predominant pathological cause of hepatic fibrosis. Our study found that serum NPY(1–36) levels were significantly higher in LC and HCC patients than in healthy volunteers. These results are similar to previous observations of plasma NPY levels, which are increased in LC patients compared to healthy controls^36^. We also found, a stepwise increase in serum NPY(1–36) as MELD scores increased and a significant positive correlation between serum NPY(1–36) levels and MELD scores in LC patients. These findings indicate a promising relationship between circulating NPY(1–36) levels and MELD scores. Moreover, serum NPY(1–36) levels in HCC patients were higher than those in healthy controls, likely because 80–90% of all cirrhotic livers develop HCC^9,10^. Ultimately, 90.57% of patients in the HCC cohort in the present study were developed from liver cirrhosis.

As previously reported, circulating NPY can be degraded head-to-toe by a variety of proteolytic enzymes, such as dipeptidyl peptidase 4, which produces NPY(3–36), meprin A, which produces NPY(2–36), and kallikreins, which produce NPY(3–35)^37,38^. Unlike most loss-of-function protein, the degradation of NPY results in a loss of affinity for the original receptor and binding to different receptors within distinct physiological responses. For example, the NPY receptor is strongly and selectively influenced by N-terminal truncation to NPY(3–36), resulting in a switch from NPY1R to NPY2R and NPY5R^37,39^. In the present study, we limited our research to NPY(1–36), mainly because of the dominant expression of NPY1R in LX-2 cells and liver tissues. Considering the abundance of proteolytic enzymes in liver and blood, we speculated that increased circulating NPY(1–36) in LC and HCC patients is partly due to a reduction in enzymes production or activity. This speculation should be tested in future studies.

A shortcoming of our study is that we have no evidence to indicate the source of the elevated serum levels of NPY(1–36) in LC and HCC patients. Histological studies have found that a large number of NPY-containing nerve fibres were distributed along with hepatocytes, sinusoid endothelial cells and Ito cells in the Disse space and that these nerve fibres were decreased in human alcoholic cirrhotic livers compared with healthy controls^17,40,41^. Combining these findings with the findings of our study, we speculated that the loss of innervation feedback stimulates the sympathetic system to increase NPY release from nerve terminals and maintain microenvironment homeostasis in the Disse space.

**Figure 6:**
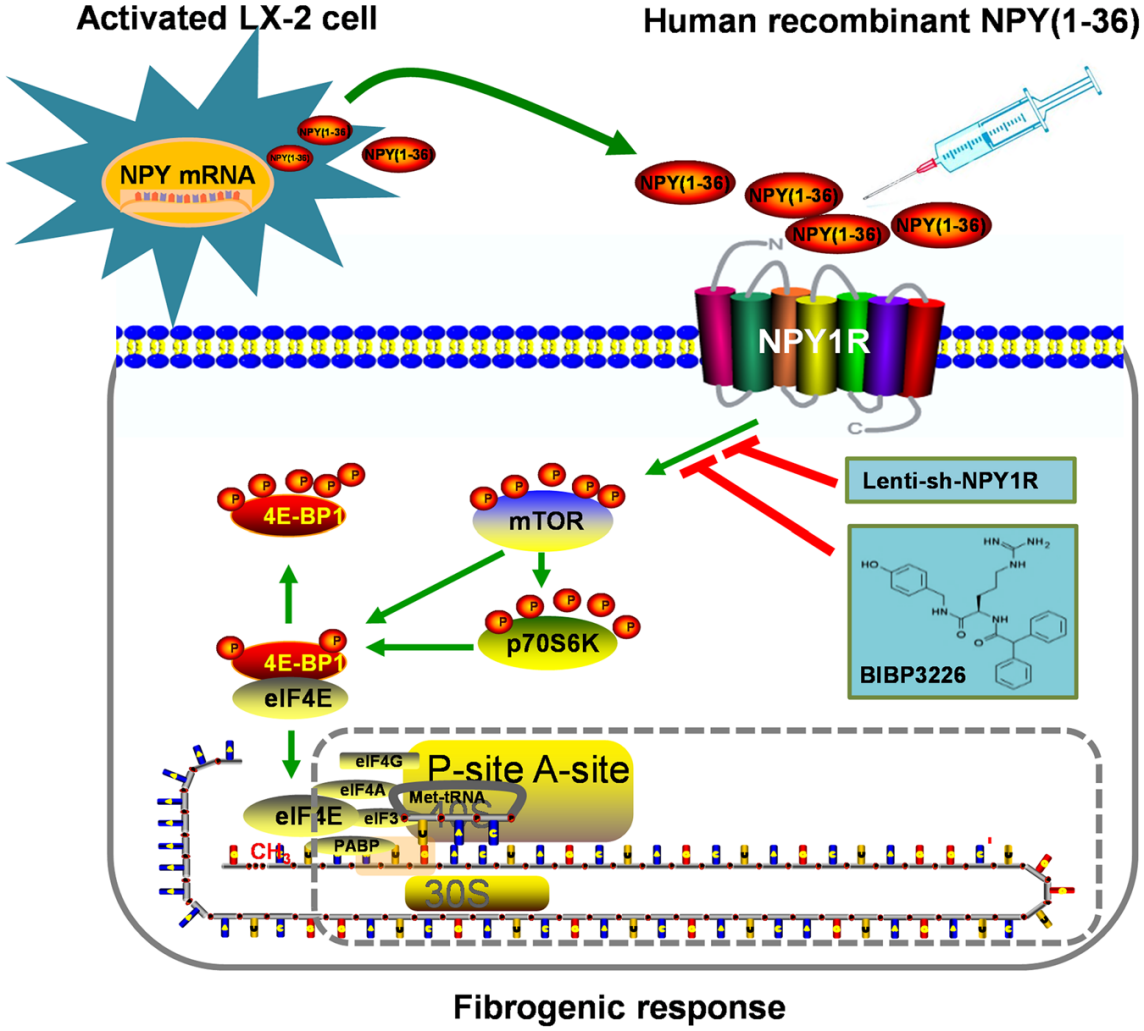
Schematic diagram of NPY(1–36) expression and secretion in activated LX-2 cells and the signalling pathway involved. Activated human immortalized hepatic stellate cells synthesize and release NPY(1–36). Both autocrine and exogenous NPY(1–36) induce fibrogenic responses via NPY1R and activate the mTOR/p70S6K/4EBP1 signalling pathway, further phosphorylating 4EBP1 and releasing eIF4E to initiate mRNA translation. The fibrogenic response can be blocked by either the selective NPY1R antagonist BIBP3226 or Lenti-sh-NPY1R. The speculated mechanism is circled with the grey dotted line.

In summary, the results of present study indicate that human immortalized hepatic stellate cell line LX-2 had the ability to produce NPY(1–36). Both autocrine and exogenous NPY(1–36) induce fibrogenic responses in LX-2 cells via NPY1R, and mTOR/p70S6K/4EBP1 signalling pathways are involved in (Fig. 6). Moreover, circulating NPY(1–36) levels are increased in LC patients and are associated with the severity of LC.

## Materials and Methods

### Cell culture and treatment

The human immortalized hepatic stellate cell line LX-2 was a gift from Dr Scott Friedman. The HCC cell line HCCLM3 was provided by the Liver Cancer Institute of Zhongshan Hospital of Fudan University (Shanghai, China). The L-02 human immortalized hepatocyte cells and the neuroblastoma cell line SH-SY5Y were purchased from the Cell Bank of Type Culture Collection of Chinese Academy of Sciences (Shanghai, China). The embryonic kidney cell line HEK293T was purchased from American Tissue Culture Collection (ATCC) (Manassas, USA). Cells were cultured in Dulbecco’s modified Eagle’s medium (DMEM, Gibco, Gaithersburg, MD, USA) containing 10% foetal bovine serum (FBS, Gibco) and incubated at 37 °C in a humidified environment containing 5% CO_2_.

To determine NPY(1–36) expression and secretion, cells were serum starved overnight and then treated with transforming growth factor β1 (TGF-β1) (#8915, Cell Signaling Technology, MA, USA) (0.1, 1, and 10 ng/mL) in the presence or absence of 10% FBS for 24 h for immunocytochemistry, western blotting and real-time PCR. For the proliferation and migration assays, the cells were serum starved overnight and then treated with recombinant human protein NPY(1–36) (#N5017, Sigma-Aldrich, USA) (10^−8^ M) or pre-treated for 2 h with the NPY1R antagonist BIBP3226 (10^−7^ M) (#60-1-22, American Peptide Company, Calif., USA). Cell proliferation and migration were assessed by the CCK-8 and transwell assays. NPY(1–36) concentrations in culture medium, cell lysates and human serum were determined by ELISA. Detailed methods are provided in the Supplementary Information.

### Plasmid constructs and lentivirus production

The plasmid constructs and lentivirus production were performed as previously described^16^. To construct the shRNAs of NPY1R in the shRNA expression vector pGreenPuro (System Biosciences, CA), three target sequences (sh1RNA, sh2RNA and sh3RNA) were synthesized and inserted into the vector according to the manufacturer’s instructions. shRNA with a non-targeting sequence was used as a negative control. Details regarding the primers are listed in Table S1.

Virus packaging was performed in HEK293T cells after the co-transfection of pGreenPuro-sh-NPY1R with the packaging plasmid pPACK-GAG, pPACK-REV (System Biosciences, CA), and the envelope plasmid pVSV-G (System Biosciences, CA) using Lipofectamine 2000 (Invitrogen, Carlsbad, CA). Viruses were harvested 48 h after transfection, and viral titres were determined. LX-2 cells (1 × 10^5^) were infected with 1 × 10^6^ recombinant lentivirus-transducing units in the presence of 6 μg/mL polybrene (Sigma) for 24 h. The LX-2 cells stably infected with recombinant lentivirus were named Lenti-sh1NPY1R, Lenti-sh2NPY1R and Lenti-sh3NPY1R (knockdown) and Lenti-vector (control).

### Immunocytochemistry

Cells were plated on glass coverslips (Fisher Scientific) in 12-well culture dishes and grown to approximately 50% confluence for 24 h to promote adherence. The cells were then fixed with 4% paraformaldehyde for 10 min and permeabilised with PBS containing 0.1% Triton X-100 for 15 min. They were then incubated with blocking solution (Beyotime) for 30 min. Samples were incubated with primary antibody (α-SMA and NPY(1–36), Sigma; NPY1R, AbCam) at 4 °C overnight and then incubated with Alexa Fluor 488 or Alexa Fluor 555 secondary antibody (Invitrogen) at room temperature for 1 h. Nuclei were stained with Hoechst 33342 (Invitrogen) for 5 min. Following a final three washes with PBS, the cells were imaged by fluorescence microscopy DMI4000B (Leica).

### Real-time polymerase chain reaction and Western blot assay

As described in our previous study^16^, total RNA was isolated from cells or tissues with TRIzol reagent (Invitrogen) according to the manufacturer’s protocol. Real-time polymerase chain reaction (PCR) was performed with the CFX96 Touch™ Real-Time PCR Detection System (Bio-Rad Laboratories, Inc.). Real-time PCR was subsequently performed with SsoFast™ EvaGreenSupermix (Bio-Rad, Shanghai, China). The reaction conditions were as follows: 95 °C for 3 min, followed by 40 cycles of 95 °C for 10 sec and 55 °C for 30 sec. The expression levels were normalized against those of the internal reference gene β-actin, and the relative expression levels were determined by the following equation: 2^−ΔΔ^Ct (^Δ^Ct = ^Δ^Cttarget^−Δ^Ctβ-actin). Primers for the target genes are listed in Table S1.

For Western blot assay, cells were prepared with the T-PER tissue protein extraction reagent (Pierce, Rockford, IL) with a cocktail of proteinase inhibitors (Roche Applied Science, Switzerland) and a cocktail of phosphatase inhibitors (Roche Applied Science). The total protein concentration was determined by the modified Bradford assay according to the manufacturer’s instructions (Sigma). Proteins were electrophoresed via SDS-PAGE and transferred to polyvinylidene difluoride (PVDF) membranes. The blots were incubated with the primary antibodies and secondary antibodies and developed using the West Dura chemiluminescent substrate. Antibodies for target proteins and incubation conditions are listed in Table S2. The bands were visualized and quantified using the ChemiDoc™ MP System (Bio-Rad). Normalization of protein α-SMA and protein phosphorylation experiments was based on the ratio to vehicle control, defined as the average (phosphorylated/total protein) for each condition divided by the corresponding average value for the control.

### Cell proliferation and migration

For proliferation assays, cells were seeded at 2 × 10^3^ cells/well in 96-well plates and cultured in 100 µl of culture medium with or without treatment with NPY(1–36) or the NPY1R antagonist BIBP3226. The corresponding culture medium was replaced every 2 days. Details were performed as previously described^16^. For migration assays, 40,000 NPY-treated or untreated LX-2 cells resuspended in 200 µl of serum-free DMEM were plated in the upper chambers (Millicell, 0.8 µm; Millipore, Bedford, MA) as previously discribed^42^. DMEM with 10% FBS was used as a chemoattractant in the lower chambers. After 16 h (for NPY(1–36) treatment) or 24 h (for NPY1R knockdown), non-migrating cells were removed from the upper surface softly by a cotton swab. The cells that migrated through the membrane to the lower surface were stained with crystal violet (Sigma-Aldrich), and then, the cells were counted and imaged using a microscope (Leica) at 100-fold magnification. This experiment was performed in triplicate.

### Cell culture supernatants and cell lysates

A total of 1 × 10^6^ cells were seeded on a 100-mm tissue culture dish and cultured to 70% confluence. The cells were serum starved and supplemented with 0.2% BSA for 48 h prior to TGF-β1 treatment (0.1, 1 and 10 ng/mL for) 24 h. Cell culture supernatants were collected, and particulates were removed by centrifugation for 15 min at 1000 × *g*, 4 °C; samples were then aliquoted and stored at −80 °C. The media was removed, and the cells were rinsed once with ice-cold PBS. The cells were scraped off the plate and transferred to a tube. The cell suspension was diluted with 1x PBS until the cell concentration reached 1 × 10^6^/mL. Then, the samples were stored at −20 °C overnight. After two freeze-thaw cycles to break up the cell membranes, cell lysates were centrifuged at 4 °C for 5 min at 5000 × *g*. The supernatants were collected, and samples aliquoted and stored at −80 °C. The samples were centrifuged again after thawing before the assay. Care was taken to avoid repeated freeze-thaw cycles.

### Human serum and the NPY(1–36) ELISA assay

We recruited consecutive patients with LC and HCC as well as healthy controls from the Affiliated Hospital of Nantong University between May 2014 and June 2017. The study was carried out according to the ethical guidelines of the 1975 Declaration of Helsinki (2008 revision) and approved by the Nantong University associated Hospital Research Ethics Committee. Written informed consent for the biological studies was obtained from each patient. There was no known disease or condition associated with serum NPY(1–36) changes in any of the participants. Diagnosis of cirrhosis was based on histopathology of liver biopsy samples. Clinicopathological characteristics of LC patients (n = 74) and HCC (n = 53) are shown in Tables S3 and S4. The model for end-stage liver disease (MELD) score was applied to assess the severity of chronic liver disease and to evaluate hepatic function in cirrhotic patients. The formula of the score is R = 9.57 × loge [creatinine (mg/dl)] + 3.78 × loge [bilirubin (mg/dl)] + 11.2 × loge (INR) + 6.43 × (aetiology: 0 if cholestatic or alcoholic, 1 otherwise)^22^. The blood donors in healthy control group have no history of liver disease, normal liver biochemistry and no malignant disease.

Peripheral blood samples were collected into serum separator tubes. Samples were allowed to clot at 4 °C overnight before centrifugation for 15 min at 1000 × *g*. Serum was collected, and samples were aliquoted and stored at −80 °C to avoid repeated freeze-thaw cycles until testing. Cell culture supernatants and cell lysates were prepared as described above. Human serum samples were diluted 50-fold with sample diluent. NPY(1–36) concentrations were measured with an enzyme-linked immunosorbent assay (ELISA) (Cusabio, China) according to the manufacturer’s recommendations. The optical density was measured at 450 nm on a microplate reader (Tecan, Austria), 570 nm was the reference wavelength. The concentrations of NPY(1–36) were calculated with a four-parameter logistic curve fitting for the standard value, and multiplied by the dilution of serum.

### Statistical analysis

Statistical analyses were carried out using the GraphPad Prism software program. For quantitative data, groups are reported as the mean ± SEM and compared using the unpaired 2-tailed t test, one-way ANOVA test or two-way ANOVA test followed with the Newman-Keuls multiple comparisons test. The linear regression and Pearson correlation were used to determine the correlationship between two variants. Statistical significance was established at p < 0.05. Unless otherwise specified, all assays were performed in triplicates.

## Supplementary information

Supplementary information
