# Supplementary material for: Expression of neuropeptide Y is increased in an activated human HSC cell line
Source: Sci Rep. 2019 Jul 1;9:9500. doi: 10.1038/s41598-019-45932-3 (PMC6602956; doi:10.1038/s41598-019-45932-3)
Supplement: Supplementary file 1 — Supplementary information [file 41598_2019_45932_MOESM1_ESM.docx]

**Supporting information for:**

Expression of neuropeptide Y is increased in an activated human HSC cell line

**Author names and affiliations**

Wufei Dai^1^, Yang Liu^1^, Yali Zhang^2^, Yufeng Sun^1^, Changjiang Sun^3^, Yu Zhang^4^ Xiufang Lv^1*^

1. Basic Medical Research Centre, Medical College of Nantong University, Nantong, China;

2. Department of Biochemistry, Medical College of Nantong University, Nantong, China;

3. Department of Clinical Laboratory, Affiliated Hospital of Nantong University, Nantong, China;

4. Department of hepatobiliary Surgery, Affiliated Hospital of Nantong University, Nantong, China;

***Corresponding author**

Xiufang Lv, MD & Ph.D., Basic Medical Research Centre in Medical College of Nantong University, No.19, Qixiu Road, Nantong, 226001, China

Tel: +86-0513-85051678; Fax: +86-0513-85051655

E-mail: [2005lxf801@163.com /](mailto:2005lxf801@163.com%20/) [Lvxiufang@ntu.edu.cn](mailto:Lvxiufang@ntu.edu.cn)


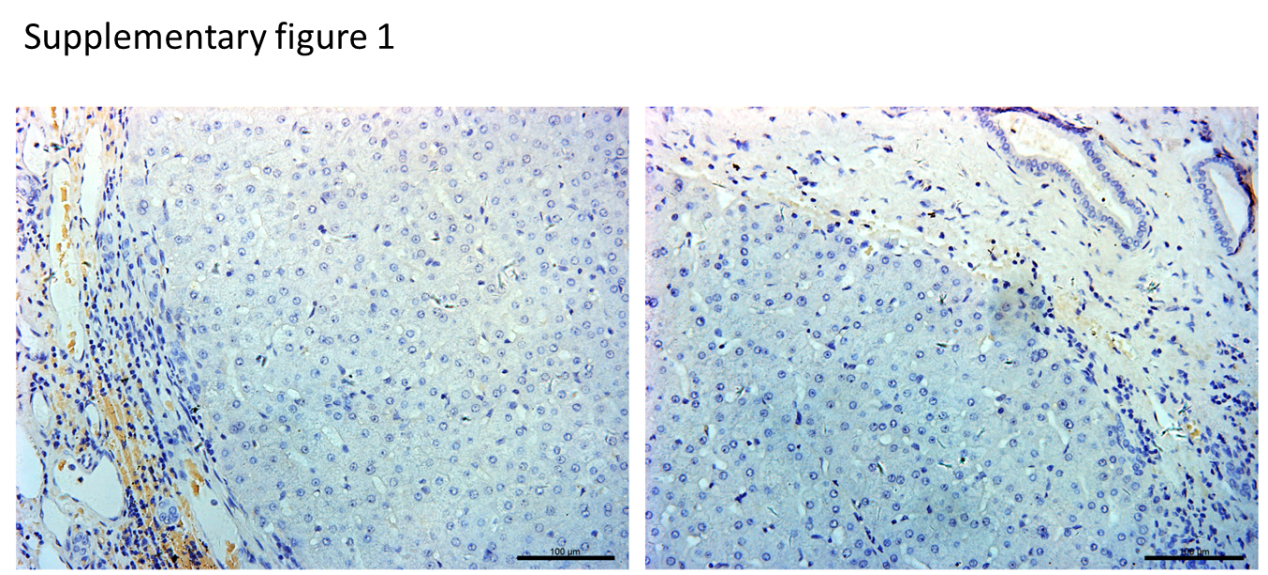


**Supplementary figure 1. Expression of NPY in liver tissues.** Representative images of NPY immunostaining in tow individual samples. Scale bars, 100 µm.

**Supporting Tables**

**Table S1. The sequences of the oligonucleotides for RT-PCR, Real-time PCR, and plasmids construct.**

| **Real-time PCR** | | | | | | |
| --- | --- | --- | --- | --- | --- | --- |
| **Gene** | **Accession No.** | | **Sequence** | **Fragment Length (bp)** | | |
| NPY | NM_000905.3 | | L: 5’- AGGACGCACCAGCGGAGGAC-3’  R: 5’- TGCAGGGTCTTCAAGCCGAGTTC-3’ | | 276 | |
| [NPY1R](http://www.ncbi.nlm.nih.gov/nuccore/NM_000909.5) | NM_000909.5 | | L: 5’- TTTGGTGAGGCGATGTGTAA-3’  R: 5’- GAAGAAGCCACAGCAAGGAC-3’ | | 188 | |
| NPY2R | NM_000910.2 | | L: 5’- CATCTTGCTTGGGGTAATTGGC-3’  R: 5’- AGAGTGAACGGTAGACACAGAG-3’ | | 212 | |
| NPY5R | NM_006174.2 | | L: 5’- GCTGGATCAGTGGATGTTTGG-3’  R: 5’- CAGATGGCAAAACCTAGTGTCC-3’ | | 247 | |
| β-actin | [NM_001101.3](http://www.ncbi.nlm.nih.gov/UniGene/seq.cgi?ORG=Hs&SID=1729733) | | L: 5’- CTCCATCCTGGCCTCGT-3’  R: 5’- GCTGTCACCTTCACCGTTCC-3’ | | 268 | |
| **Plasmid Construct** | | | | | | |
| [NPY1R](http://www.ncbi.nlm.nih.gov/nuccore/NM_000909.5) | NM_000909.5 | Sense:5'-GATCCGAAACCTGGCCTTGATCATAACTTCCTGTCAGATTATGATCAAGGCCAGGTTTCTTTTTG-3'  Anti-sense:5'-AATTCAAAAAGAAACCTGGCCTTGATCATAATCTGACAGGAAGTTATGATCAAGGCCAGGTTTCG-3' | | | | sh1RNA |
| [NPY1R](http://www.ncbi.nlm.nih.gov/nuccore/NM_000909.5) | NM_000909.5 | Sense:5'-GATCCCGGACTCTCATAGGTTGTCTTCTTCCTGTCAGAAAGACAACCTATGAGAGTCCGTTTTTG-3'  Anti-sense:5'-AATTCAAAAACGGACTCTCATAGGTTGTCTTTCTGACAGGAAGAAGACAACCTATGAGAGTCCGG-3' | | | | sh2RNA |
| [NPY1R](http://www.ncbi.nlm.nih.gov/nuccore/NM_000909.5) | NM_000909.5 | Sense:5'-GATCCGCTCCCTCTTACCATCTTTAACTTCCTGTCAGATTAAAGATGGTAAGAGGGAGCTTTTTG-3'  Anti-sense:5'-AATTCAAAAAGCTCCCTCTTACCATCTTTAATCTGACAGGAAGTTAAAGATGGTAAGAGGGAGCG-3' | | | | sh3RNA |

**Table S2. The proteins detected and the characteristics of the corresponding antibodies**

| **Protein** | **Assay** | **Origin** | **Dilution** | **Incubation period** |
| --- | --- | --- | --- | --- |
| NPY | ICC | N9528，Sigma-Aldrich, Inc. Saint Louis, USA | 1:500 | Overnight |
| α-SMA | ICC/WB | A5228, Sigma-Aldrich, Inc. Saint Louis, USA | 1:400/  1:1000 | Overnight |
| NPY1R | WB  ICC | sc-28949, Santa Cruz Biotechnology. Inc, Santa Cruz, CA  ab91262，Cambridge, MA, USA | 1:1000  1：500 | Overnight  Overnight |
| phospho-mTOR (Ser2448) | WB | cs-5536, Cell Signaling Technology. Inc, Beverly, MA | 1:1000 | Overnight |
| mTOR (7C10) | WB | cs-2983, Cell Signaling Technology. Inc, Beverly, MA | 1:1000 | Overnight |
| Phospho-p70 S6 Kinase (Ser371) | WB | cs-9208, Cell Signaling Technology. Inc, Beverly, MA | 1:1000 | Overnight |
| Phospho-p70 S6 Kinase (Thr389) | WB | cs-9234, Cell Signaling Technology. Inc, Beverly, MA | 1:1000 | Overnight |
| p70 S6 Kinase | WB | cs-9202, Cell Signaling Technology. Inc, Beverly, MA | 1:1000 | Overnight |
| Phospho-4E-BP1 (Thr37/46) | WB | cs-2855, Cell Signaling Technology. Inc, Beverly, MA | 1:1000 | Overnight |
| 4E-BP1 | WB | cs-9425, Cell Signaling Technology. Inc, Beverly, MA | 1:1000 | Overnight |
| β-actin (8H10D10) | WB | sc-3770, Santa Cruz Biotechnology. Inc, Santa Cruz, CA | 1:5000 | Overnight |

**Table S3: The clinicopathologic characteristics of patients with liver cirrhosis (LC) patients**

| Variable | | Test | | |  |
| --- | --- | --- | --- | --- | --- |
|  |  | No. | Percentage(%) | |  |
| Age, years |  |  | |  |  |
|  | Mean | 52.39 | | |  |
|  | SD | 9.71 | | |  |
| Gender |  |  |  | |  |
|  | Female | 10 | 13.51 | |  |
|  | Male | 64 | 86.49 | |  |
| AFP (ng/ml) |  |  |  | |  |
|  | ≤20 | 47 | 63.51 | |  |
|  | ＞20 | 27 | 36.49 | |  |
| HBsAg |  |  |  | |  |
|  | Negative | 16 | 21.62 | |  |
|  | Positive | 58 | 78.38 | |  |
| HBeAg |  |  |  | |  |
|  | Negative | 54 | 72.97 | |  |
|  | Positive | 20 | 27.03 | |  |
| HBsAb |  |  |  | |  |
|  | Negative | 66 | 89.19 | |  |
|  | Positive | 8 | 10.81 | |  |
| HBeAb |  |  |  | |  |
|  | Negative | 55 | 74.32 | |  |
|  | Positive | 19 | 25.68 | |  |
| GGT |  |  |  | |  |
|  | ＜54 (U/L) | 21 | 28.38 | |  |
|  | ＞54 (U/L) | 53 | 71.62 | |  |
| MELD |  |  |  | |  |
|  | ≤10 | 35 | 47.29 | |  |
|  | 10-20 | 31 | 41.89 | |  |
|  | 20-30 | 8 | 10.81 | |  |
| Aetiology |  |  |  | |  |
|  | Posthepatic* | 58 | 78.38 | |  |
|  | Primary biliary | 7 | 9.46 | |  |
|  | Parasitic | 5 | 6.76 | |  |
|  | Alcoholic | 4 | 5.40 | |  |
| **Abbreviations:** AFP=alpha-fetoprotein. HBsAg=hepatitis B surface antigen. HBsAb=hepatitis B surface antibody. HBeAg=hepatitis B e antigen. HBeAb=hepatitis B e antibody. GGT, Gamma-Glutamyl Transferase  *, Posthepatitic: cirrhosis caused by HBV. | | | | | |

**Table S4: The clinicopathologic characteristics of patients with HCC**

| Variable | | Test | |
| --- | --- | --- | --- |
|  |  | No. | Percentage (%) |
| Age, years |  |  |  |
|  | Mean | 51.17 | |
|  | SD | 10.45 | |
| Gender |  |  |  |
|  | Female | 12 | 22.64 |
|  | Male | 41 | 77.36 |
| AFP (ng/ml) |  |  |  |
|  | ≤20 | 9 | 16.98 |
|  | ＞20 | 44 | 83.02 |
| Liver cirrhosis |  |  |  |
|  | no | 5 | 9.43 |
|  | yes | 48 | 90.57 |
| HBsAg |  |  |  |
|  | Negative | 11 | 20.76 |
|  | Positive | 42 | 79.24 |
| HBeAg |  |  |  |
|  | Negative | 38 | 71.70 |
|  | Positive | 15 | 28.30 |
| GGT |  |  |  |
|  | ＜54 (U/L) | 11 | 20.76 |
|  | ＞54 (U/L) | 42 | 79.24 |
| Tumor differentiation |  |  |  |
|  | I-II | 26 | 49.06 |
|  | III-IV | 27 | 50.94 |
| TNM stage |  |  |  |
|  | I-II | 25 | 47.17 |
|  | III | 28 | 52.83 |
| **Abbreviations:**  AFP=alpha-fetoprotein. HBsAg=hepatitis B surface antigen. HBeAg=hepatitis B e antigen. GGT, Gamma-Glutamyl Transferase | | | |

| **Table S5. Correlation of clinicpathological characteristics in health control and patients** | | | | | |
| --- | --- | --- | --- | --- | --- |
| Variable | | Patients (n) | | | *P* value |
|  |  | HC | LC | HCC |  |
| Age, years | ≤50 years | 18 | 25 | 15 | 0.5678 |
|  | ＞50 years | 29 | 49 | 38 |  |
| Gender | Female | 8 | 10 | 12 | 0.4055 |
|  | Male | 39 | 64 | 41 |  |
| NPY(ng/ml) | ≤20 | 24 | 20 | 9 | **0.0008** |
|  | ＞20 | 23 | 54 | 44 |  |
| AFP (ng/ml) | ≤20 | / | 47 | 9 | **<0.0001** |
|  | ＞20 | / | 27 | 44 |  |
| HBsAg | Negative | / | 16 | 11 | 0.9063 |
|  | Positive | / | 58 | 42 |  |
| HBeAg | Negative | / | 54 | 38 | 0.1160 |
|  | Positive | / | 20 | 15 |  |
| GGT | ＜54 (U/L) | / | 21 | 11 | 0.3291 |
|  | ＞54 (U/L) | / | 53 | 42 |  |
| Chi-square (and Fisher's exact) test was used.  The bolding stands for the p-values with signiﬁcant difference.  **Abbreviations:** HC=healthy individuals; LC=liver cirrhosis; HCC=hepatocellular carcinoma. | | | | | |
